# VIRTUAL CLINICAL TRIALS IN MEDICAL IMAGING SYSTEM EVALUATION AND OPTIMISATION

**DOI:** 10.1093/rpd/ncab080

**Published:** 2021-06-18

**Authors:** Bruno Barufaldi, Andrew D A Maidment, Magnus Dustler, Rebecca Axelsson, Hanna Tomic, Sophia Zackrisson, Anders Tingberg, Predrag R Bakic

**Affiliations:** Department of Radiology, University of Pennsylvania, 3400 Spruce Str., Philadelphia, PA 19104, USA; Department of Radiology, University of Pennsylvania, 3400 Spruce Str., Philadelphia, PA 19104, USA; Department of Translational Medicine, Lund University, Skane University Hospital, Carl-Bertil Laurells gata 9, Malmö 20502, Sweden; Department of Translational Medicine, Lund University, Skane University Hospital, Carl-Bertil Laurells gata 9, Malmö 20502, Sweden; Department of Translational Medicine, Lund University, Skane University Hospital, Carl-Bertil Laurells gata 9, Malmö 20502, Sweden; Department of Translational Medicine, Lund University, Skane University Hospital, Carl-Bertil Laurells gata 9, Malmö 20502, Sweden; Department of Translational Medicine, Lund University, Skane University Hospital, Carl-Bertil Laurells gata 9, Malmö 20502, Sweden; Department of Radiology, University of Pennsylvania, 3400 Spruce Str., Philadelphia, PA 19104, USA; Department of Translational Medicine, Lund University, Skane University Hospital, Carl-Bertil Laurells gata 9, Malmö 20502, Sweden

## Abstract

Virtual clinical trials (VCTs) can be used to evaluate and optimise medical imaging systems. VCTs are based on computer simulations of human anatomy, imaging modalities and image interpretation. OpenVCT is an open-source framework for conducting VCTs of medical imaging, with a particular focus on breast imaging. The aim of this paper was to evaluate the OpenVCT framework in two tasks involving digital breast tomosynthesis (DBT). First, VCTs were used to perform a detailed comparison of virtual and clinical reading studies for the detection of lesions in digital mammography and DBT. Then, the framework was expanded to include mechanical imaging (MI) and was used to optimise the novel combination of simultaneous DBT and MI. The first experiments showed close agreement between the clinical and the virtual study, confirming that VCTs can predict changes in performance of DBT accurately. Work in simultaneous DBT and MI system has demonstrated that the system can be optimised in terms of the DBT image quality. We are currently working to expand the OpenVCT software to simulate MI acquisition more accurately and to include models of tumour growth. Based on our experience to date, we envision a future in which VCTs have an important role in medical imaging, including support for more imaging modalities, use with rare diseases and a role in training and testing artificial intelligence (AI) systems.

## INTRODUCTION

Virtual Clinical Trials (VCTs) in medical imaging have been used to design, evaluate and optimise imaging systems, to prototype clinical trials and for regulatory approval. VCTs are used as a rapid and cost-effective alternative to conducting some clinical trials, allowing researchers to answer fundamental questions using *in silico* simulations.

Researchers at the University of Pennsylvania (UPenn) developed a VCT framework that encompasses the use of computer models of human anatomy, imaging modalities and image interpretation^(1)^. In 1998, an anthropomorphic breast model was developed to support simulations of breast imaging^(^^2^^,^  ^3^^)^, and by 2009, a software framework to design and optimise breast imaging systems using VCTs was completed^(4–10)^. Numerous academic laboratories^(^^11^^,^  ^12^^)^, industrial developers^(^^13^^,^  ^14^^)^ and governmental regulatory bodies^(15)^ have since adopted VCTs. Several VCT use-cases have been published, including the evaluation and optimisation of digital breast tomosynthesis (DBT)^(16–22)^, breast and lung computed tomography (CT)^(23–25)^, denoising of breast X-ray images^(^^26^^,^  ^27^^)^ and dermatology imaging^(^^28^^,^  ^29^^)^.

Today, VCTs are accepted as an efficient preclinical optimisation tool. Based on our experience in designing and conducting VCTs, in this paper, we review VCT principles and major simulation components. The benefits and challenges of VCTs are illustrated through our new results in the assessment of lesion detection in breast imaging and the review of our recently published design and optimisation of simultaneous DBT and mechanical imaging (MI) of the breast. (The breast lesion detection results presented here have been significantly expanded from our preliminary published report^(16)^).

## OPENVCT SIMULATION FRAMEWORK

The OpenVCT framework^(30)^ has been used to generate anthropomorphic breast models and simulate breast positioning, mammographic compression, X-ray image acquisition and human interpretation. The breast anatomy is simulated using an octree-based recursive partitioning algorithm^(5)^, where random seeds are used to direct the simulation of glandular and adipose compartments bounded by fibrous Cooper’s ligaments. The mammographic compression uses finite element (FE) software, which deforms the breast models in accordance with clinical breast views^(31)^. The image acquisition is simulated by a ray-tracing algorithm^(30)^.

Human readings are simulated using the MeVIC software^(32)^ (Barco Healthcare), which is integrated into the OpenVCT framework. The MeVIC software is designed to simulate a high-resolution medical display, then process and analyse the displayed images based upon a Channelized Hotelling Observer (CHO) model^(33)^. The display simulation includes details of the video card and monitor, including the greyscale lookup table and luminance characteristics, temporal characteristics, modulation transfer function, noise and angular dependence^(^^34^^,^  ^35^^)^. The CHOs can be trained and tested repeatedly to simulate human readers and that can be used to estimate multiple-reader multiple-case (MRMC) ROC statistics^(36)^.

The execution of every simulation step and the data synchronisation between the simulation steps and between the OpenVCT and MeVIC software occur via a configurable XML schema and are recorded as a multipart compressed file (VCTx). The VCTx files contain the prescribed XML files and the input and output binary documents. The format was designed to optimise data exchange and storage for VCTs^(30)^.

## STUDY I: VCTS FOR SYSTEM EVALUATION

### Methodology

Together, the OpenVCT and MeVIC software were used to evaluate lesion detectability in DM and DBT. Our preliminary results were published in 2018^(16)^. This study compared simulation results with published clinical data from two 2013 studies by Rafferty *et al*.^(37)^. A commercial DM and DBT system was simulated. Masses were simulated with an ellipsoidal shape, diameter of 7 mm and thickness of 0.5–2 mm. Single microcalcifications were simulated as containing 1–4 voxels of size (100 μm)^3^. Detection of lesions was simulated using CHOs. Performance of the machine observers was assessed by the MRMC ROC analysis. The VCTs were calibrated by selecting a set of simulated lesions to match the clinical lesion detectability with DM images. The lesions were varied in terms of size and attenuation.

Our study presented here expanded the previous analysis by detailed selection of simulated lesions to achieve close matching of the VCT with clinical DM results. Performance of the machine observers was assessed in detail by the MRMC ROC analysis. For each of the two clinical studies^(37)^, an admixture of simulated lesions was used to match the clinical performance to DM only in terms of the shape and area under the ROC curve (AUC). The same sets of simulated lesions were then used to generate synthetic DBT images, and the simulated detectability was compared with the clinical DBT data.

### Results and discussion

**Figure 1:**
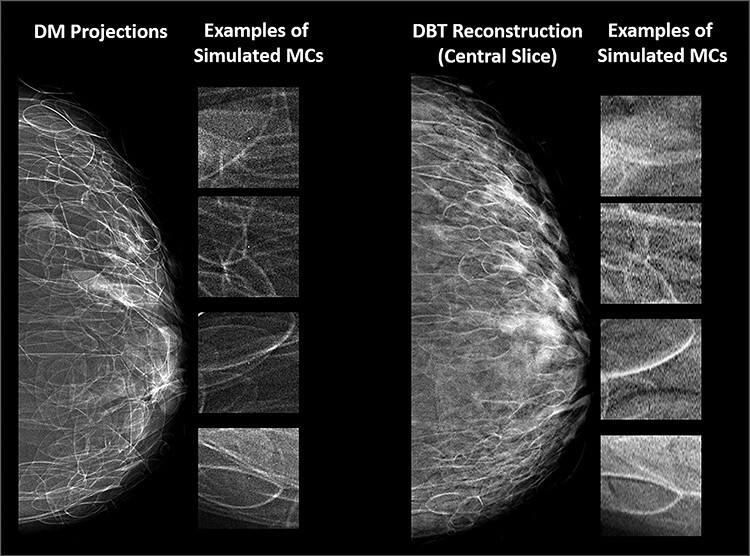
examples of synthetic breast images with simulated microcalcifications, generated using OpenVCT software.

**Figure 2:**
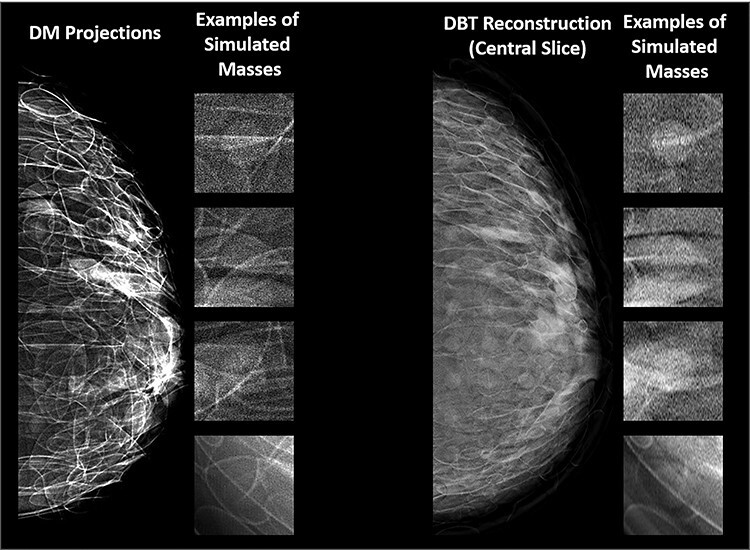
examples of synthetic breast images with simulated masses, generated using OpenVCT software.

**Figure 3:**
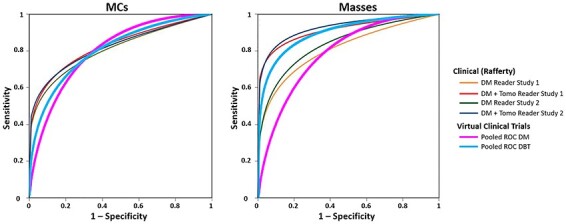
ROC curves of clinical and virtual lesion detectability in DM and DBT, fitted properly for the MRMC analysis using the ROC + KIT software (University of Chicago).

Examples of synthetic breast images with simulated microcalcifications and masses are shown in Figures 1 and 2. In this study of lesion detectability, close agreement between the virtual and clinical estimates of detectability (Figure 3) is observed. The AUCs, estimated from the VCTs and the published reader study, are tabulated for the detection of microcalcifications (Table 1) and masses (Table 2). Tables 1 and 2 also include the AUC differences and their corresponding *p*-values and 95% confidence intervals (CIs). The difference between the AUC values from VCTs and the published work was less than 4%, demonstrating the ability of VCTs to predict human reader performance.

The use of VCTs in this task had specific advantages. Using the OpenVCT framework, the computational effort to replicate the reader study as a VCT took less than 4 d on a single GPU card (P5000, NVidia Corp, Santa Clara, CA). By comparison, the original study took significantly more time and was much costlier. However, as noted in Figure 3, differences still exist between the shape of the ROC curves of the VCT and clinical trials, demonstrating a challenge for VCTs. We are currently working to fine-tune the virtual population and virtual lesions to better match the shape of the ROC curves from the VCTs and the clinical data.

## STUDY II: VCTS FOR SYSTEM OPTIMISATION

### Methodology

Currently, we are investigating methods to extend the use of VCTs to multimodality imaging, specifically to design, evaluate and optimise the prototype of a simultaneous DBT and MI system (DBTMI)^(^^38^^,^  ^39^^)^. Combining the radiographic DBT characterisation of anatomy with the functional measurement of local tissue stiffness using MI is expected to increase cancer detection and reduce the rate of false positives, improving the specificity of cancer detection^(^^38^^,^  ^39^^)^.

To explore the relationship between sensitivity and specificity of the two constituent modalities (DBT and MI), together with their combined performance, we expanded the OpenVCT framework to include simulations of the MI. MI acquisition is simulated with the FE models of the breast tissue and mammographic compression^(40)^. A preliminary study^(40)^ used linear elastic material models of the breast tissue, a spherical tumour approximation and tumour stiffness of 15–50 times the stiffness of the simulated breast tissue. The open source software, FEBio^(41)^, was used to calculate the stress on the surface of the compressed breast and to simulate the response of the MI sensor (BRE 5350-2, Tekscan, Boston, MA). The simulated response was compared with the clinical values^(^^42^^,^  ^43^^)^. A detailed description of the MI simulation and preliminary evaluation was presented at the 2021 SPIE Medical Imaging conference paper by Axelsson *et al*.^(40)^.

A model of tumour growth is also under development to evaluate DBTMI. Modelling tumour growth can identify optimal screening intervals, aimed at reducing interval cancers. To model tumour growth, we simulated two screening exams of the same breast at the time of the cancer detection and at the prior screening round either 18 or 24 months earlier (following the protocol from the Swedish Screening Programme^(44)^).

The OpenVCT framework was used to simulate 30 breasts, with one spherical tumour in each breast^(45)^. Tumour growth was modelled by increasing the tumour volume exponentially over time according to the tumour volume doubling time (TVDT)^(^^46^^,^  ^47^^)^. The woman’s age, tumour diameter at the time of cancer detection and TVDT value were selected from the clinical data^(46)^. A radiologist manually indicated tumour diameters on the simulated mammograms. The diameters of the same lesions at the two time points were used to calculate the estimated TVDT. The growth model was evaluated by comparing TDVTs from the simulated mammograms with the clinical data. The growth model and its evaluation in an observer study were described by Tomic *et al*.^(45)^.

### Results and discussion

**Figure 4:**
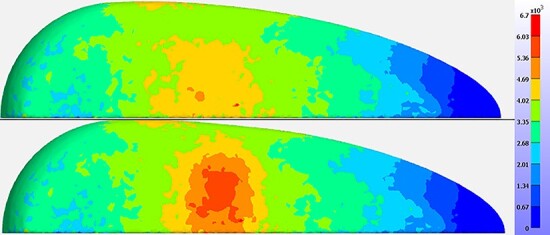
distribution of surface stress generated using FE software, FEBio, with simulated spherical tumours of two different sizes: 4 mm (top) and 7.5 mm (bottom).

Figure 4 shows two stress surface maps obtained using the FE model for a virtual breast with spherical lesions of different sizes. The average stress over the virtual breast surface (6.2 ± 0.1 kPa) is in agreement with the average stress measured clinically^(42)^ (5.6 ± 2.0 kPa). The average stress at the simulated tumour (10.8 ± 6.4 kPa) is higher than the clinically reported values^(43)^ (6.8 ± 5.3 kPa). The simulation is affected by the tissue model. To improve the agreement, we are currently investigating the use of hyperelastic tissue models.

**Table 1:** Result of the ROC analysis for detection of breast microcalcifications using DM and DBT, from virtual and clinical data. Listed are the AUC values estimated from synthetic breast images generated using OpenVCT, the AUC values from two clinical studies performed by Dr. Rafferty (denoted 1 and 2), the AUC differences and the corresponding *p*-values and 95% CIs.

Microcalcifications	AUC_DM_	AUC_DBT_	ΔAUC_DBT–DM_	*p*	95% CI
VCT	0.802 ± 0.023	0.799 ± 0.026	−0.003	0.856	[−0.040, 0.034]
Clinical^(37)^					
1	0.804	0.840	0.036	0.073	[−0.004, 0.074]
2	0.817	0.831	0.014	0.082	[−0.002, 0.029]
AUC difference VCT versus clinical					
1	−0.002	−0.041			
2	−0.015	−0.032			

**Table 2:** Result of the ROC analysis for detection of breast masses using DM and DBT, from virtual and clinical data. Listed are the AUC values estimated from synthetic breast images generated using OpenVCT, the AUC values from two clinical studies performed by Dr. Rafferty’s (denoted 1 and 2), the AUC differences and the corresponding *p*-values and 95% CIs.

Masses	AUC_DM_	AUC_DBT_	ΔAUC_DBT–DM_	*p*	95% CI
VCT	0.794 ± 0.022	0.900 ± 0.017	0.106	<0.001	[0.089, 0.124]
Clinical^(37)^					
1	0.807	0.912	0.105	<0.001	[0.047, 0161]
2	0.842	0.930	0.088	<0.001	[0.051, 0.125]
AUC difference VCT versus clinical					
1	−0.013	−0.012			
2	−0.048	−0.03			

**Figure 5:**
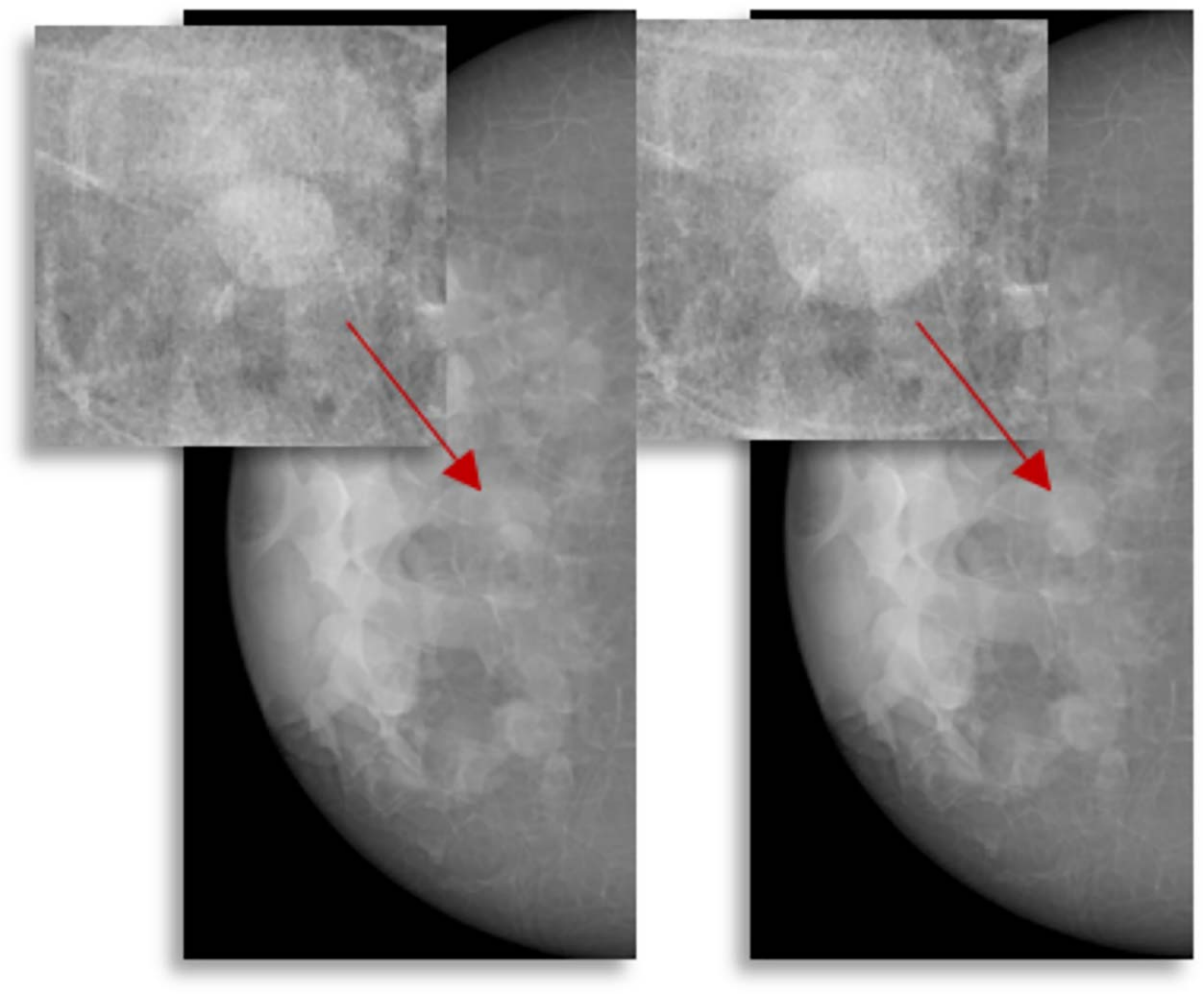
synthetic DM images of two consecutive screening episodes, containing a simulated tumour (arrows) with doubling time of 374 d.

Figure 5 shows screening mammograms simulated 24 months apart, assuming a spherical lesion model with a TVDT of 374 d. The tumour sizes in the current and prior simulated mammograms were 11. and 7.2 mm, respectively. The sizes measured by the radiologist were 12.2 and 7.6 mm, respectively, corresponding to a TVDT of 356 d (18 d or 4.8% lower than the ground truth). Analysing all 30 simulated breasts, no significant difference was seen between the estimated and ground-truth TVDTs, with a median difference of 12 d (4%) (*p* > 0.5 using the Kolmogorov–Smirnov test)^(45)^.

The limitations of the growth model include the spherical tumour shape and the lack of changes in the background breast tissue between the two simulated screening exams. To overcome these limitations, we are currently developing a model for irregularly shaped tumours. In addition, the temporal changes in the normal breast tissue will be simulated using an analysis of clinical images over multiple screening rounds.

## CONCLUSIONS

Today, VCTs of medical imaging are sufficiently mature to play an important role in the design of imaging systems and the validation of clinical trials, for use by engineers, physicians and regulatory authorities. The open-source OpenVCT framework software has been extensively validated as demonstrated and reviewed here. In a comparison of lesion detectability in DM and DBT, VCTs could accurately rank the imaging system performance and predict the level of performance improvement. In addition, VCTs played an important role in designing and optimising a novel combined imaging method, DBTMI.

Based upon our experience with VCTs, we continue to expand the OpenVCT framework. This includes work to calibrate the VCT observer studies to clinical studies by fine-tuning the virtual population and virtual lesions. We are also modelling tumour growth to relate equipment performance differences into the potential for screening benefits to women.

Looking at the future, there are several directions in which VCTs can benefit medical imaging. First, rare and paediatric diseases are characterised by a small number of patients and limited amount of available clinical images, which impedes the clinical research^(48)^. VCTs can play an important role once models of such diseases are available. Second, the demand for training and testing datasets for AI in medical imaging has increased. Synthetic images generated by VCTs may represent an efficient and cost-effective option to augment and expand clinical data. Given the rapid development of VCTs in medical imaging, we envision them to have a growing role in the future.
